# Analysis of amino acid motif of penicillin-binding proteins 1a, 2b, and 2x in invasive *Streptococcus pneumoniae* nonsusceptible to penicillin isolated from pediatric patients in Casablanca, Morocco

**DOI:** 10.1186/s13104-018-3719-5

**Published:** 2018-08-31

**Authors:** Idrissa Diawara, Kaotar Nayme, Khalid Katfy, Abouddihaj Barguigua, Mohamed Kettani-Halabi, Houria Belabbes, Mohammed Timinouni, Khalid Zerouali, Naima Elmdaghri

**Affiliations:** 1 Laboratoire de Microbiologie, Faculté de Médecine et de Pharmacie, Hassan II University of Casablanca, B.P 5696, Casablanca, Morocco; 2 Service de Microbiologie, CHU Ibn Rochd, B.P 2698, Casablanca, Morocco; 3 Mohammed VI University of Health Sciences (UM6SS), Casablanca, Morocco; 4 Molecular Bacteriology Laboratory, Institut Pasteur du Maroc, Casablanca, Morocco; 5 Laboratory of Biotechnology and Sustainable Development of Natural Ressources, Polydisciplinary Faculty, Université Sultan Moulay Slimane, Beni Mellal, Morocco

**Keywords:** *Streptococcus pneumoniae*, Penicillin-binding proteins, β-lactams, Serotypes, Penicillin resistance, *pbp* gene

## Abstract

**Objectives:**

This study aimed to investigate the nature of the amino acid motifs found in PBPs of *Streptococcus pneumoniae* isolates in invasive diseases from pediatric patients at Casablanca, Morocco. Five penicillin-susceptible (PSSP), ten penicillin-intermediate (PISP), and fifteen penicillin-resistant *S. pneumoniae* (PRSP) were studied by PCR–RFLP and DNA sequencing of the *pbp1a*, − *2b*, and − *2x* genes.

**Results:**

There were no changes in the conserved motifs of PBP1a, PBP2b and PBP2x for PSSP strains. Substitution close to PBP1a conserved motifs were found in all PRSP isolates and six/five PISP. Analysis of PBP2b showed that all but one of the 10 PISP strains and all PRSP had substitutions. Substitution close to PBP2x motifs showed that all but three of the 10 PISP strains and all PRSP had substitutions in tow conserved motifs. A total of 6, 11 and 10 genotypes were found after analysis of *pbp1a*, *pbp2b*, and *pbp2x*, respectively. The penicillin-nonsusceptible *S. pneumoniae* isolated in Casablanca share most amino acid substitutions of those reported worldwide, but they occurred among pneumococci with low level resistance to b-lactams.

## Introduction

*Streptococcus pneumoniae* is worldwide a common cause of invasive diseases such as meningitis, bacteraemia [1]. The treatment of pneumococcal infection has been compromised due to the acquisition of several antibiotic resistance, especially to β-lactam drugs [2, 3]. Resistance to β-lactams has been associated in pneumococcus, to alterations of the penicillin-binding proteins (PBP) which reduce their affinity [4]. β-Lactam antibiotics exert their biological effects by interacting with the PBPs. Resistance in clinical pneumococci to this antibiotic family is associated mainly to the alteration of PBP1a, PBP2b and PBP2x [5]. The active site of these PBPs is formed by three conserved amino acid motifs, SXXK, SXN, and KT(S)G. These motifs are found at amino acid positions 370–373, 428–430, and 557–559 in PBP1a, at positions 337–340, 395–397, and 547–549 in PBP2x, and at positions 385–388, 442–444, and 614–616 in PBP2b [4]. Changes in these motifs, or in the positions flanking, are associated with low-affinity variants of the PBPs. It has been previously described that penicillin resistance in *S. pneumoniae* is mediated by stepwise alterations of PBPs [6, 7]. Several studies have described the genetic profile of the three major *plp* genes: *pbp1a*, *pbp2b* and *pbp2x* genes in *S. pneumoniae* around the world. But currently, there are no data on penicillin resistant *S. pneumoniae* in Morocco. Furthermore, given that the proportion of penicillin resistance in *S. pneumoniae* has considerably increased in Morocco since 1998 [8], and Casablanca is part of this emerging trend, we described the nature of the *pbp1a*, *pbp2b* and *pbp2x* amino acid motifs in 30 clinical pneumococci isolated from pediatric patients in Casablanca.

## Main text

### Materials and methods

#### Bacterial strains, growth conditions and DNA extraction

Thirty invasive pneumococcal isolates: 5 penicillin-susceptible *S. pneumoniae* (PSSP): MIC ≤ 0.06 mg/L, 10 penicillin-intermediate *S. pneumoniae* (PISP): > 0.06 and < 2 mg/L, and 15 penicillin-resistant *S. pneumoniae* (PRSP): MIC ≥ 2 mg/L, were randomly chosen (from 2007 to 2016) to represent the different ranges of MICs of *S. pneumoniae* found IPD in Casablanca. Antibiotic susceptibility testing was done following Clinical Laboratory Standard Institute guidelines 2016, with the same antibiotics and the same methods used in our previously study [3].

Pneumococci were obtained from the bacteria bank of the laboratory of Microbiology of the University hospital centre of Casablanca, Morocco. The procedure employed for bacterial growth and capsular typing was previously described [9].

For DNA extraction, we use QIAamp^®^DNA mini kit (QIAGEN, Valencia, CA) according to manufacturer’s recommendations.

#### PCR–RFLP and DNA sequencing of pbp1a, − 2b, and − 2x genes

Genetic polymorphism of the penicillin resistance genes *pbp1a*, *pbp2b*, and *pbp2x* was investigated by restriction fragment length polymorphism (RFLP) analysis as described previously [3, 10]. As previously studied [3], the different *pbp* genotypes received a three numbers code (e.g., x/y/z) referring to the RFLP patterns of the genes *pbp1a* (x), *pbp2b* (y), and *pbp2x* (z), respectively. The 5 penicillin-susceptible *S. pneumoniae* were used as positive control. Both strands of the purified amplicons (ExoSAP-IT™ PCR, ThermoFisher Scientific, Carlsbad, CA, USA) were sequenced with a Genetic Analyzer 3130x1 sequencer (Applied Biosystems, Foster City, CA, USA), with the same primers used for PCR amplification as recommended by the Centers for Disease Control and Prevention (CDC, USA) [11]. The results of DNA sequencing were aligned using ChromasPro software version 1.7 (Technelysium Pty. Australia) and Basic Local Alignment Search Tool available on Internet at the National Center for Biotechnology Information website. The nucleotide and derived amino acid sequence data for strains are compared to the corresponding sequence data for the β-lactam susceptible laboratory isolate R6 (sequence available at GenBank Accession Numbers: *pbp1a*M90527; *pbp2b*X16022; *pbp2x*X16367).

## Results

Clinical isolates from various patients were chosen for this study. The age of the patients from whom the isolates were recovered ranged from 0.1 to 5 years. The geometric mean values of MICs (and MIC ranges) in milligrams per liter for penicillin, amoxicillin and ceftriaxone for the three groups with their serotypes were presented in the Table 1. Co-resistance rates among the isolates showed that, 60% of PISP were nonsusceptible to tetracycline, 50% to erythromycin, and 40% to cotrimoxazole. As for PRSP, 86.6% were nonsusceptible to cotrimoxazole, 20% to tetracycline and erythromycin, and 13% to chloramphenicol **(**Table 1). There was no co-resistance for PSSP (wild profile).

**Table 1:** Origins, characteristics and pattern of resistance of the 30 pneumococcal isolates tested in this study

Strain no.	Sources	Age (year)	Year of isolation	Serotype	MIC (mg/L)			Resistance profile
					PG	AMX	CRO	
Penicillin-susceptible								
1	Blood	0.3	2014	1	0.008	0.003	0.008	Wild
2	CSF	0.3	2013	8	0.016	0.016	0.016	Wild
3	CSF	1	2012	9V	0.016	0.016	0.008	Wild
4	Blood	0.1	2011	2	0.016	0.016	0.008	Wild
5	PF	0.7	2010	6B	0.016	0.016	0.016	Wild
Penicillin-intermediate								
6	Blood	0.1	2013	6B	0.125	0.06	0.004	PG, Ery, TE
7	CSF	0.4	2008	23F	0.125	0.032	0.064	PG
8	Blood	5	2008	6A	0.125	0.125	0.25	PG, Ery
9	Blood	4	2007	19F	0.125	0.25	0.25	PG, SXT, TE
10	Blood	3	2007	22F	0.25	0.064	0.032	PG
11	CSF	3	2012	14	0.5	0.25	0.25	PG, SXT
12	Pus	4	2011	19A	0.5	0.5	0.25	PG, Ery, TE
13	CSF	0.6	2013	6B	1	0.12	0.25	PG, Ery, TE
14	CSF	3	2010	6B	1	2	0.5	PG, Ery, SXT, TE
15	Blood	4	2014	14	1	1	0.5	PG, SXT, TE
Penicillin-resistant								
16	Blood	0.5	2009	14	2	1	1	PG, SXT
17	CSF	0.5	2010	14	2	2	1	PG, SXT
18	CSF	0.3	2010	14	2	4	0.25	PG, SXT
19	PF	3	2011	14	2	1	0.5	PG, Ery, SXT, TE
20	CSF	1	2009	23F	2	4	0.5	PG, Ery, SXT, Chl, TE
21	Blood	0.3	2007	14	2	2	0.5	PG, SXT
22	Blood	3	2013	14	2	1	0.5	PG
23	Blood	0.6	2011	14	2	2	0.5	PG
24	Blood	1	2007	14	2	2	0.75	PG, SXT
25	Pus	0.8	2009	6B	2	2	0.5	PG, Ery, SXT, Chl, TE
26	Blood	0.5	2013	14	2	0.5	0.5	PG, SXT
27	Blood	0.4	2010	9V	2	4	0.5	PG, SXT
28	Blood	1	2009	14	2	2	0.5	PG, SXT
29	CSF	1.3	2010	14	2	2	0.5	PG, SXT
30	Blood	3	2011	14	2	1	0.25	PG, SXT

*CSF* cerebrospinal fluid, *PF* pleural fluid, *MIC* minimal inhibitory concentration, *PG* penicillin G, *AMX* amoxicillin, *CRO* ceftriaxone, *SXT* cotrimoxazole, *TE* tetracycline, *Ery* erythromycin, *Chl* chloramphenicol

The amino acid sequences of the three conserved PBP motifs of the three PBP studied and their genotypic profiles are shown in Table 2. There were no changes in the conserved motifs of PBP1a, PBP2b and PBP2x for PSSP.

**Table 2:** PBP1a, PBP2b and PBP2x genetic profiles of the 30 pneumococcal isolates in Casablanca, Morocco

Strain no.	Penicillin-binding protein motifs									*pbp* profile		
	PBP1a			PBP2b			PBP2x			*plp1a*	*plp2b*	*plp2x*
	STMK (370–373)	SRNVP (428–432)	KTG (557−559)	SVVK (385–388)	SNNT (442–445)	KTGTA (614–618)	STMK (337–340)	HSSN (395–397)	LKSG (546–549)			
Penicillin-susceptible												
1	−−−−	−−−−	−−−−	−−−−	−−−−	−−−−−	−−−−	−−−−	−−−−	5	11	7
2	−−−−	−−−−−	−−−	−−−−	−−−−	−−−−−	−−−−	−−−−	−−−−	5	1	7
3	−−−−	−−−−−	−−−	−−−−	−−−−	−−−−−	−−−−	−−−−	−−−−	5	2	7
4	−−−−	−−−−−	−−−	−−−−	−−−−	−−−−−	−−−−	−−−−	−−−−	5	2	7
5	−−−−	−−−−−	−−−	−−−−	−−−−	−−−−−	−−−−	−−−−	−−−−	5	2	7
Penicillin intermediate												
6	−−−−	−−−−−	−−−	−−−−	−−−A	−−−−−	−A−−	−−−−	V−−−	5	10	7
7	−−−−	−−−−−	−−−	−−−−	−−−A	−−−−−	−A−−	−−−−	V−−−	4	5	9
8	−A−−	−−−−−	−−−	−−−−	−−−A	−−−−−	−−−−	−−−−	−−−−	6	8	8
9	−A−−	−−−−T	−−−	−−−−	−−−−	−−−−−	−−−−	L−−−	−−−−	3	2	4
10	−−−−	−−−−−	−−−	−−−−	−−−A	−−−−−	−−−−	−−−−	−−−−	5	9	3
11	−A−−	−−−−T	−−−	−−−−	−−−A	−−−−−	−A−−	−−−−	V−−−	1	4	6
12	−A−−	−−−−T	−−−	−−−−	−−−A	−−−−−	−A−−	−−−−	V−−−	2	7	1
13	−−−−	−−−−−	−−−	−−−−	−−−A	−−−−−	−A−−	−−−−	V−−−	5	3	2
14	−A−−	−−−−T	−−−	−−−−	−−−A	−−−−G	−A−−	−−−−	V−−−	2	6	7
15	−A−−	−−−−T	−−−	−−−−	−−−A	−−−−−	−A−−	−−−−	V−−−	1	4	6
Penicillin resistant												
16	−A−−	−−−−T	−−−	−−−−	−−−A	−−−−−	−A−−	−−−−	V−−−	2	4	6
17	−A−−	−−−−T	−−−	−−−−	−−−A	−−−−−	−A−−	−−−−	V−−−	2	4	6
18	−A−−	−−−−T	−−−	−−−−	−−−A	−−−−G	−A−−	−−−−	V−−−	1	8	5
19	−A−−	−−−−T	−−−	−−−−	−−−A	−−−−−	−A−−	−−−−	V−−−	2	4	10
20	−A−−	−−−−T	−−−	−−−−	−−−A	−−−−−	−A−−	−−−−	V−−−	1	4	6
21	−A−−	−−−−T	−−−	−−−−	−−−A	−−−−−	−A−−	−−−−	V−−−	2	4	1
22	−A−−	−−−−T	−−−	−−−−	−−−A	−−−−−	−A−−	−−−−	V−−−	1	4	5
23	−A−−	−−−−T	−−−	−−−−	−−−A	−−−−−	−A−−	−−−−	V−−−	2	2	2
24	−A−−	−−−−T	−−−	−−−−	−−−A	−−−−−	−A−−	−−−−	V−−−	2	4	7
25	−A−−	−−−−T	−−−	−−−−	−−−A	−−−−−	−A−−	−−−−	V−−−	2	6	7
26	−A−−	−−−−T	−−−	−−−−	−−−A	−−−−−	−A−−	−−−−	V−−−	1	4	6
27	−A−−	−−−−T	−−−	−−−−	−−−A	−−−−−	−A−−	−−−−	V−−−	1	6	6
28	−A−−	−−−−T	−−−	−−−−	−−−A	−−−−−	−A−−	−−−−	V−−−	1	4	1
29	−A−−	−−−−T	−−−	−−−−	−−−A	−−−−−	−A−−	−−−−	V−−−	2	4	6
30	−A−−	−−−−T	−−−	−−−−	−−−A	−−−−−	−A−−	−−−−	V−−−	1	1	1

*S*: serine, *T*: threonine, *M*: methionine, *K*: lysine, *R*: arginine, *N* asparagine, *V* valine, *P* proline, *G* glycine, *A* alanine, *H* histidine, *L* leucine, *PBP* penicillin-binding protein

As for the amino acid alterations of the three conserved motifs of all PNSP, no mutation is reported close to the KTG (PBP1a) and SVVK (PBP2b) conserved motifs.

Substitution close to PBP1a conserved motifs showed Thr^371^ → Ala substitution in the conserved STMK motif in all PRSP and six PISP, Pro^432^ → Thr (SRNVP motif) in all PRSP and five PISP.

Analysis of PBP2b showed that 96% of PNSP had Thr^445^ → Ala substitutions in the SNNT motif. Substitution close to PBP2x motifs showed that all but three of the 10 strains of PISP and all PRSP had Thr^338^ → Ala substitutions in the STMK motif and had Leu^546^ → Val substitutions close to the LKSG motif.

The Ala^618^ → Gly substitution close to the third PBP2b conserved motif (KTG) was identified in only one PISP and one PRSP strain (Table 2). The only change close to the motifs SSN in the PBP2x was a His^394^ → Leu substitution. This change was found in only one strain with MIC to PG at 0.125 mg/L.

**Fig. 1:**
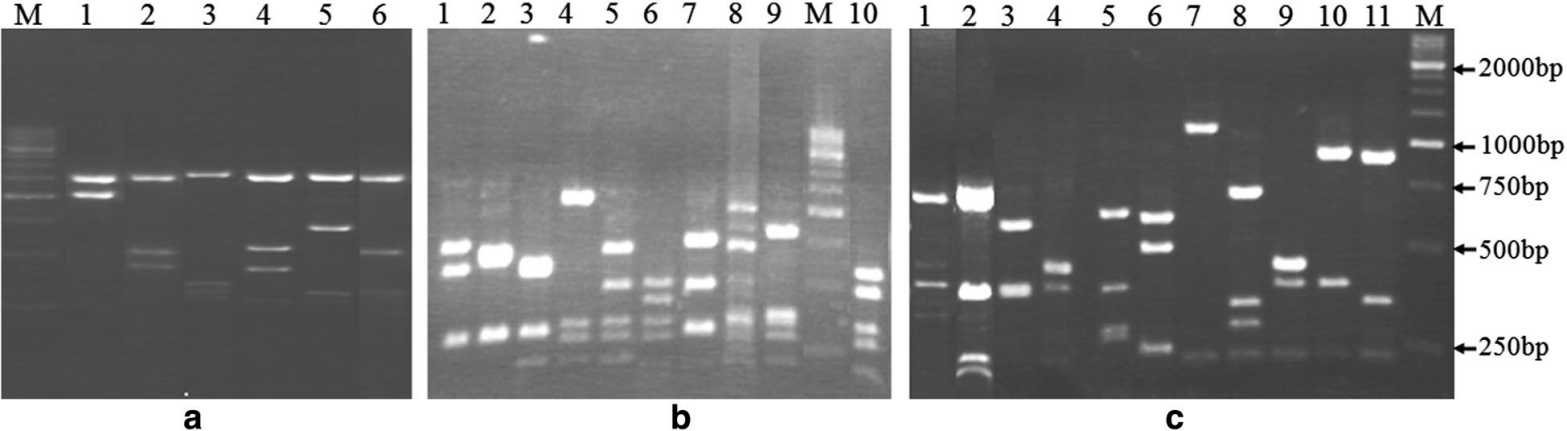
PCR–RFLP profiles of the different *pbp* genes digested by *Hae*III and *Rsa*I. **a** Profile of *pbp1a* gene, lanes 1–6 correspond to RFLP profiles; **b** profile of *pbp2x* gene, lanes 1–10 correspond to RFLP profiles. **c** Profile of *pbp2b* gene digested, lanes 1–11 correspond to RFLP profiles. Line M corresponds to DNA size standards (250 base pairs)

A total of 6, 11 and 10 restriction profiles were found after analysis of *pbp* gene by PCR–RFLP specific for *pbp1a*, *pbp2b*, and *pbp2x*, respectively (Fig. 1). Twenty-four different composite pattern profiles for the three resistance genes were found among the 30 isolates. There were 3, 9 and 12 different composite pattern profiles for PSSP, PISP and PRSP strains, respectively.

We found only one profile for *pbp1a* and *pbp2x* while *pbp2b* presented three different profiles for PSSP strains (Table 2). Concerning the PISP and PRSP strains, there were a several genotypes for the three genes. However, among the PRSP strains, we found only two types of profile for *pbp1a* gene and the major composite profile was 2/4/6 present in three strains with serotype 14 and 1/4/6 in two strains (one serotype 14 and one serotype 23F).

## Discussion

PBPs are the major resistance determinants in the pneumococcus. The low-affinity variants of PBPs are the results of recombination of the genes coding for these proteins with genes of other species, such as viridans streptococci. Previous studies have suggested that PBP1a, − 2x, and − 2b are generally recognized as the major PBPs associated with the activities of penicillins and some cephalosporins [4, 12]. In our study, changes found in PBP1A, PBP2b and PBP2X are globally similar to those previously reported [6, 13, 14].

The maximal level of resistance of PNSP responsible of invasive pneumococcal disease reported in Casablanca, is relatively low with the maximal MICs = 2 mg/L compared to MICs of PNSP in many countries where MICs ≥ 8 mg/L were reported [14, 15]. Our investigation of the PBP1a, − 2x, and − 2b amino acid sequences of 30 clinical pneumococci demonstrates that the degree of diversity within these amino acid sequences correlates with increasing resistance to β-lactam antibiotics. Analysis of PBP1a, − 2x, and − 2b penicillin-binding motifs revealed the absence of substitution in or close to the active site of all motif analyzed in PSSP strains in this study. These findings differed from previously studies. Indeed Nagai et al. are found Thr^445^ → Ala substitutions close to SNNT motif in PBP2b gene and Leu^546^ → Val substitutions close to the LKSG motif in PBP2x gene in PSSP strains [6]. Granger et al. are also found Thr^445^ → Ala substitutions in the SNNT motif in PBP2b in one PSSP strain in Canada [16]. It is not clear how some PSSP isolates can harbor these two mutations without becoming non-susceptible to penicillin. PSSP analyzed in this study are probably associated with a limited number of clones according to the RFLP profiles of the three *pbp* genes.

Analysis of PBP1a and PBP2b motifs revealed the absence of substitution in or close to the active site of conserved KTG and SVVK motifs. These findings are in agreement with results from other studies, suggesting that these motifs are not involved in the development of penicillin resistance [17, 18].

Interestingly, PISP isolates with amoxicillin MICs ≥ 0.125 mg/L and MICs ≥ 0.25 mg/L harbored amino acid substitutions close to PBP1a conserved motifs STMK (Thr^371^ → Ala) and SRNVP (Pro^432^ → Thr), respectively. This result suggests that alteration in conserved motif of PBP1a may be occurred among *S. pneumoniae* with low level resistance to penicillin and amoxicillin. The diversity of the pattern of amino acid motifs in the PNSP as well as *pbp2b* and *pbp2x* genes suggests these isolates have emerged independently as previously described [19].

For PRSP strains, they shared a similar pattern of amino acid motifs but had different genotypes of the three *pbp* genes. All of these isolates harbored the same amino acid substitutions close to PBP1a, PBPB2b and PBP2x conserved motifs. Similar results were published by Zhou et al. in China [14]. In addition, one strain had Ala^618^ → Gly substitutions close to KTGTA motif in the PLP2B. These changes are identical to those previously reported [6, 13]. However, we reported in this study, amino acid alteration among PNSP with low-levels of MICs (2 mg/L). In several study, amino acid alteration, especially for PBP1a, is reported for high-level penicillin resistance MICs > 4 mg/L [16, 18, 19]. Our explanation for this difference is the origin of our isolates. Indeed, non-invasive pneumococcal isolates frequently have a higher prevalence and high-levels of antimicrobial non-susceptibility, compared to invasive isolates, but they can share the same amino acid alterations.

Moreover, we found that all PNSP strains had generally some co-resistances associated with other antibiotics families especially to cotrimoxazole, tetracycline and erythromycin as reported elsewhere [20].

PCR–RFLP analysis of the *pbp1a*, *pbp2b* and *pbp2x* genes yielded six, eleven and ten distinct fingerprint patterns, respectively. Genotype 5/2/7 was found most frequently among PSSP isolates and there was a single genotype for *plp1a* and *pbp2x*. In contrast, genotype of PNSP strains, showed several composite pattern profiles for the three resistance genes. Variations in the RFLP patterns demonstrate the highly variable nature of the *pbp* genes, suggesting a high frequency recombination or point mutations that they undergoes over the time [21].

## Conclusions

This study constitutes the first investigation of *pbp* gene alterations in invasive *S. pneumoniae* isolates in Morocco. Our study reveals that penicillin-nonsusceptible *S. pneumoniae* isolated among children in Casablanca share most PBP1a, PBP2b and PBP2x amino acid substitutions with those reported worldwide. Alteration of PBP reported here occured among pneumococci with low level resistance to b-lactams. Surveillance of antibiotic-resistant pneumococci in Casablanca should be continued, with due attention to the mechanisms of resistance.

## Limitation of the study

The development of resistance to β-lactams is a complex mechanism and can be influenced by mutations in other *pbp* and non-*pbp* genes. In our study, the main limitation was that the substitutions outside the specific areas of *pbp* genes were not examined. These substitutions might also contribute to resistance, in addition to other mechanisms, in the activities of β-lactams in pneumococci [6, 7].

## Abbreviations

PBP: penicillin binding protein
PSSP: penicillin-susceptible *Streptococcus pneumoniae*
PISP: penicillin-intermediate *Streptococcus pneumoniae*
PRSP: penicillin-resistant *Streptococcus pneumoniae*
S: serine
T: threonine
M: methionine
K: lysine
R: arginine
N: asparagine
V: valine
P: proline
G: glycine
A: alanine
H: histidine
L: leucine
